# Identification of *Cryptosporidium viatorum* XVa subtype family in two wild rat species in China

**DOI:** 10.1186/s13071-019-3763-6

**Published:** 2019-10-28

**Authors:** Yi-Wei Chen, Wen-Bin Zheng, Nian-Zhang Zhang, Bin-Ze Gui, Qiu-Yan Lv, Jia-Qi Yan, Quan Zhao, Guo-Hua Liu

**Affiliations:** 1 Hunan Provincial Key Laboratory of Protein Engineering in Animal Vaccines, College of Veterinary Medicine, Hunan Agricultural University, Changsha, 410128 Hunan People’s Republic of China; 2 State Key Laboratory of Veterinary Etiological Biology, Key Laboratory of Veterinary Parasitology of Gansu Province, Lanzhou Veterinary Research Institute, Chinese Academy of Agricultural Sciences, Lanzhou, 730046 Gansu People’s Republic of China; 3 College of Life Sciences, Changchun Sci-Tech University, Shuangyang, 130600 Jilin People’s Republic of China; 4 Hunan Co-Innovation Center of Animal Production Safety, Changsha, 410128 Hunan People’s Republic of China

**Keywords:** *Cryptosporidium viatorum*, Cryptosporidiosis, Zoonosis, Wild rats, China

## Abstract

**Background:**

*Cryptosporidium viatorum* is a minor *Cryptosporidium* pathogen in humans. Currently, there is limited information regarding the prevalence and genotypes of *C. viatorum* in animals in China.

**Methods:**

In this study, 228 faecal samples were collected from two wild rat species (*Leopoldamys edwardsi* and *Berylmys bowersi*) in Chongqing Municipality and Guangdong Province, China. These specimens were analyzed for *C. viatorum* and then subtyped it using PCR and sequence analysis of the small subunit ribosomal RNA (*SSU* rRNA) and 60-kilodalton glycoprotein (*gp60*) genes, respectively.

**Results:**

A total of 25 (11.0%) faecal samples were tested positive for *C. viatorum* by *SSU* rRNA assay. Of these samples, 4 (3.6%) came from *L. edwardsi* and 21 (18.0%) from *B. bowersi*. Of the 25 *C. viatorum*-positive samples, 17 were successfully amplified at the *gp60* gene locus, which represented four subtypes belonging to two subtype families, including XVa (XVaA6, XVaA3g, XVaA3h) and XVc (XVcA2G1). Phylogenetic analysis based on the gp60 amino acid sequences indicated that all of the *C. viatorum* isolates grouped together, supporting the conclusion that *C. viatorum* from the wild rats represent two subtype families.

**Conclusions:**

These results indicate an occurrence of *C. viatorum* XVa subtype family from rats which is genetically identical to those found in humans. Our findings suggest that wild rats may be a potential source of human cryptosporidiosis.
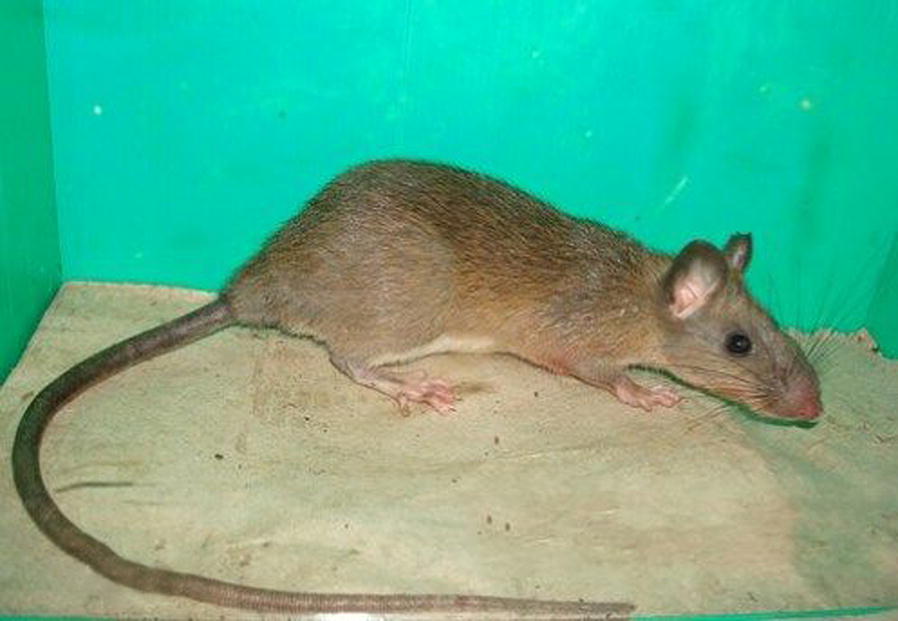

## Background

Cryptosporidiosis is caused by *Cryptosporidium* spp. and is a serious enteric disease of humans and animals worldwide [1, 2]. Humans can become infected by ingesting food and water contaminated with *Cryptosporidium* oocysts [3]. To date, more than 78 species/genotypes of *Cryptosporidium* are known to infect humans and animals [4]. *Cryptosporidium viatorum* is a novel parasite that has emerged in the last decade, and is a minor *Cryptosporidium* species to infect humans [5].

*Cryptosporidium viatorum* was first found in the faecal samples of travellers returning to Britain from the Indian subcontinent in 2012 [6]. Later, *C. viatorum* was found in two Swedish patients in 2013 [7]. Since then, *C. viatorum* infection has been recorded in patients with underdeveloped or impaired immunity (including children and AIDS patients) living in developing countries such as Nigeria, Ethiopia, Colombia and India [8–13]. Recently, there was also one case of *C. viatorum* found in western Australian human populations (2015–2018) [14]. The clinical symptoms of *C. viatorum* infection in humans include diarrhoea, fever, headache, abdominal pain, nausea, vomiting and marked weight loss [1].

There have been reports of *C. viatorum* infection (XVa subtype family) in humans, but very limited information is available regarding *C. viatorum* infection in animal hosts [15, 16]. Recently, Koehler et al. [16] reported *C. viatorum* infection (XVb subtype family) in Australian swamp rats, suggesting that it is endemic to native rats in Australia. In addition, the identification of *C. viatorum* in urban wastewater and combined sewer overflows indicated that this human pathogen may be common in China as well [17]. Very recently, Zhao et al. [15] found that *C. viatorum* (XVc and XVd subtype families) was prevalent (7.3%) in wild rats in Hainan Province of China. However, to date, it is unknown whether *C. viatorum* XVa subtype family which is in humans is present in animal hosts, including in wild rats. Wild rodents are one of the largest of the mammalian groups [18]. They have a wide distribution and can transmit numerous pathogens to other animals and to humans [19].

In China, *Leopoldamys edwardsi* and *Berylmys bowersi* are two common rat species with a wide distribution. To determine whether *C. viatorum* XVa subtype family which is in humans is present in *L. edwardsi* and *B. bowersi*, we performed an investigation of the prevalence and the subtypes of *C. viatorum* in these two wild rat species in China.

## Methods

### Specimen collection and host identification

From November 2017 to January 2018, 111 Edwardʼs long-tailed rats (*L. edwardsi*) were collected from Chongqing Municipality, China (28°10′–32°13′N, 105°11′–110°11′E) and 117 Bower’s white-toothed rat (*B. bowersi*) were collected from Guangdong Province, China (20°13′–25°31′N, 109°39′–117°19′E). A total of 228 faecal samples from these rats were separately collected and stored in 2.5% potassium dichromate solution at − 20 °C for future analysis.

Specific identification of the two wild rat species was determined by PCR-based sequencing of the mitochondrial *cox*1 gene based on a previous method [20]. The obtained sequences were compared with sequences in GenBank using the BLASTn algorithm. The *cox*1 sequences of the two wild rat species were found to have a sequence identity of more than 99% to previously published sequences for *L. edwardsi* and *B. bowersi* from China and Vietnam (GenBank: KM434322 and JN105105, respectively).

### DNA extraction and genotyping of *C. viatorum*

Fresh faecal specimens were subjected to direct DNA extraction using the E.Z.N.A.^®^ Stool DNA Kit (Omega Biotek Inc., Norcross, GA, USA) according to the manufacturer’s protocols. The extracted DNA was stored at − 20 °C for subsequent molecular analysis. Nested PCR was used to amplify and sequence regions of the small subunit ribosomal RNA (*SSU* rRNA) gene from *Cryptosporidium* spp. [21]. The 60-kilodalton glycoprotein (*gp60*) gene was used to classify the subtype levels of *C. viatorum* [22]. Positive and negative controls were included in each amplification. Amplification products were examined using 1.5% agarose gel containing GoldView^TM^ (Solarbio, China) and observed under UV light.

### Sequence and phylogenetic analysis

Positive PCR products were sent to Sangon Biotech (Shanghai, China) for sequencing in both directions. The sequences were aligned with known reference sequences available on GenBank using the Basic Local Alignment Search Tool (BLAST). The genotypes of *C. viatorum* were identified using Clustal X 1.83 [23]. Available gp60 amino acid sequences of *C. viatorum* were aligned using MAFFT 7.122 [24], and ambiguous sites and regions were excluded using Gblocks 0.91b with default parameters [25]. Phylogenetic analysis was performed using MEGA 5.0 [26]. The p-distance model was selected as the most suitable one. Neighbor-joining (NJ) tree was calculated based on 1000 bootstrap replicates.

### Statistical analysis

Statistical analysis was performed using SPSS V20.0 (IBM, Chicago, IL, USA). The following variables were analysed using the Chi-square test: variation in the prevalence of *C. viatorum* (*y*), wild rat species (*x*1), gender (*x*2) and different geographic region (*x*3). Each variable was included in a Binary Logit Model and as an independent variable in a multivariate regression analysis. The best model was judged by Fisher’s scoring algorithm. All tests were two-sided. *P *< 0.05 was considered statistically significant. Odds ratios (ORs) and 95% confidence intervals (95% CIs) were estimated to explore the strength of the association between *C. viatorum*-positivity and the test conditions.

## Results

Of the 228 wild rats collected, 25 (11.0%) were found to be *C. viatorum*-positive based on PCR amplification of the partial, small subunit (*SSU*) rRNA gene (Table 1). The *SSU* rDNA sequences from the two wild rat species exhibited more than 99.4% identity to previously published sequence from a human in Kenya (GenBank: JX978271). The overall prevalence of *C. viatorum* in *L. edwardsi* was 3.6% (4/111) and in *B. bowersi* it was 18.0% (21/117). The prevalence of *C. viatorum* in female rats (8.9%) was somewhat lower than in male rats (13.3%), although this difference was not statistically significant (*P *> 0.05).

**Table 1:** Prevalence (in %) and factors risk of *Cryptosporidium viatorum* infection in wild rats in China

Factor	Category	No. tested	No. positive	Prevalence (95% CI)	OR (95% CI)	*P*-value
Region	Chongqing	111	4	3.6 (0.14–7.1)	Reference	
	Guangdong	117	21	18.0 (11.0–24.9)	5.9 (1.9–17.7)	0.001
Gender	Female	113	10	8.9 (3.6–14.1)	Reference	0.40
	Male	115	15	13.3 (6.9–19.2)	1.6 (0.7–3.6)	
Species	*Leopoldamys edwardsi*	111	4	3.6 (0.1–7.1)	Reference	
	*Berylmys bowersi*	117	21	18.0 (11.0–24.9)	5.9 (1.9–17.7)	0.001
Total		228	25	11.0 (6.9–15.0)		

*Cryptosporidium viatorum* was subtyped by *gp60* gene sequence analysis and 17 of the 25 specimens were successfully amplified. Further analysis of these sequences suggests that four subtypes belonged to two subtype families, including XVa (XVaA6, *n* = 2; XVaA3g, *n* = 7; and XVaA3h, *n* = 7) and XVc (XVcA2G1, *n* = 1). The XVaA6 (two *L. edwardsi*) had 100% homology to that of the XVaA6 subtype from wastewater in China (GenBank: KX190061) [17]. Other three subtypes (XVaA3g, XVaA3h and XVcA2G1) were identified in the 15 *B. bowersi*. The XVaA3g had 100% homology to that of the XVaA3g subtype from human in Western Ausralia (GenBank: MK165991) [14]. Single nucleotide changes were observed between XVaA3g and XVaA3h. The XVcA2G1 had 99.2% identity with the XVcA2G1a (GenBank: MK433562) subtype identified from China.

**Fig. 1:**
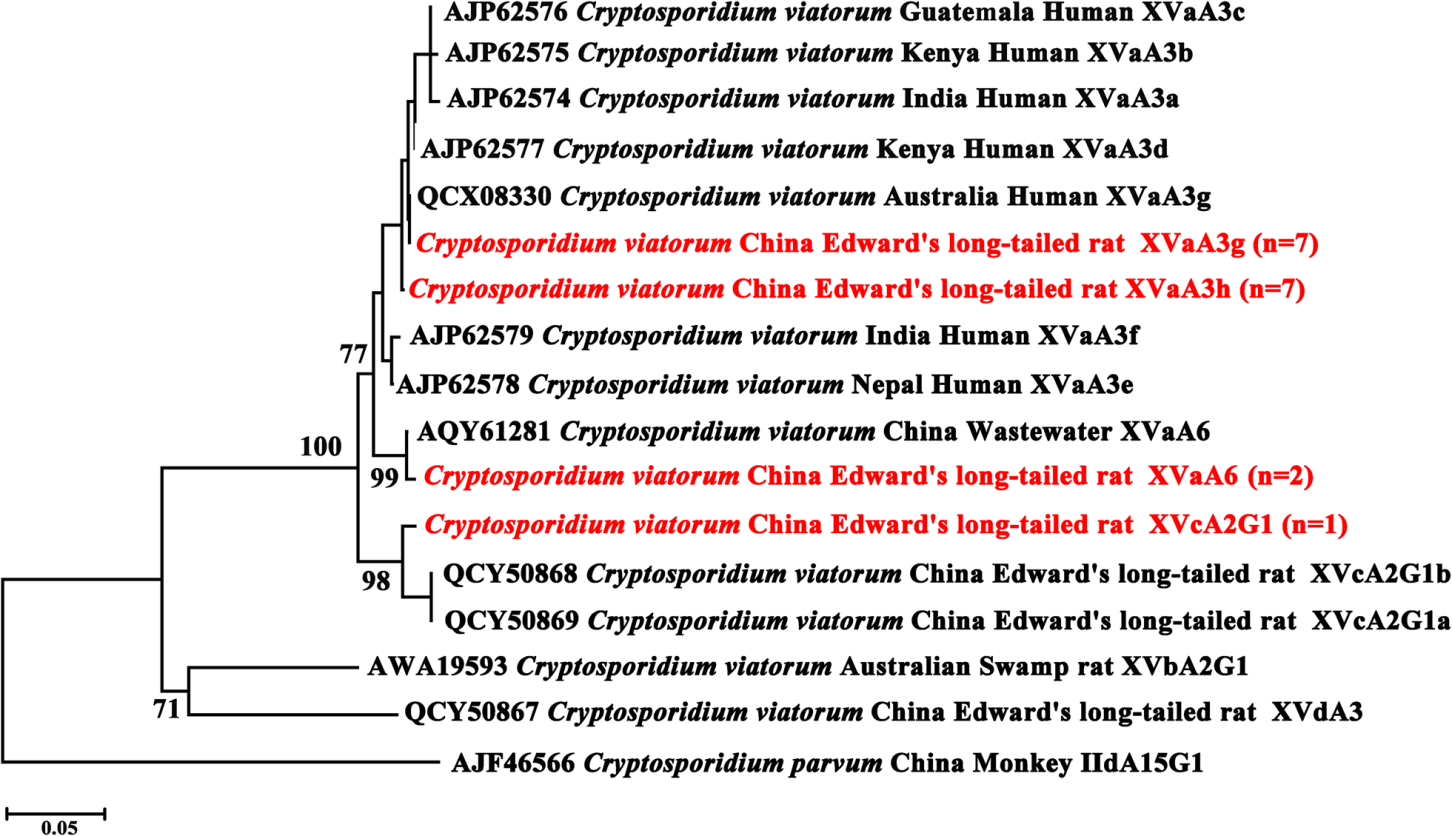
Phylogenetic analyses of gp60 amino acid data of *C. viatorum* and selected *Cryptosporidium* taxa using neighbor-joining (NJ) method

Phylogenetic analysis based on *SSU* rDNA sequences demonstrated that all of the *C. viatorum* isolates (including human isolates) grouped together with high statistical support (bootstrapping frequencies = 100) (not shown), indicating that all of the isolates from the present study represent *C. viatorum*. In addition, the *gp60* gene provided clear evidence that the present four subtypes (XVaA6, XVaA3g, XVaA3h and XVcA2G1) of *C. viatorum* from the wild rats represented two subtype families (XVa and XVc) with strong bootstrap support (Fig. 1).

## Discussion

*Cryptosporidium viatorum* has been documented in many countries [6–13], and infection can cause persistent gastrointestinal diseases in humans. The prevalence of *C. viatorum* in patients may be caused by their lack of natural or acquired resistance, as well as by behavioural and habitual activities related to environmental and socio-economic determinants [10]. Recently, *C. viatorum* was found in rats in Australia [16] and Hainan Province of China [15], indicating that these rats can act as a natural host. In the present study, *C. viatorum* was found in wild rats in China, which warrants further consideration into whether wild rats will become a potentially important avenue for *C. viatorum* transmission to humans and other animals.

The *gp60* subtyping tool has been used to divide *Cryptosporidium* into 15 subtype families (numbered I-XV) according to their *gp60* sequence [21, 27]. Stensvold et al. in 2015 [22] has classified a number of *C. viatorum* sequences isolated from humans into six subtypes (XVaA3a-XVaA3f). Recently, a new subtype (XVaA3g) of *C. viatorum* has been found in an Australian patient [14]. To our knowledge, to date, only the XVa subtype family has found in *C. viatorum*-positive patients [22] and wastewater in China [17]. Interestingly, the results of the present study provide clear evidence that the three subtypes (XVaA6, XVaA3g and XVaA3h) from the wild rats belong to the XVa subtype family which is genetically identical to those found in humans, suggesting that wild rats may have a potential for zoonotic transmission, and must be considered as a potential threat to human health. Our results indicate an occurrence of *C. viatorum* XVa subtype family in wild rats, indicating that *C. viatorum* XVa subtype family may be an extensive host range. In fact, in previous studies, XVa subtype family of *C. viatorum* were not found in rats (Edwardʼs long-tailed rats and Australian swamp rats) [15, 16]. However, we should be cautious whether the finding of XVa subtype family in rats represents a natural infection needs to be further confirmed with additional datasets. Unfortunately, we are unable to determine the source of infection and transmission dynamics of *C. viatorum* XVa subtype family in investigated rats due to the lack of *C. viatorum* data from humans in the investigated areas. In addition, three novel subtypes (XVcA2G1a, XVcA2G1b and XVdA3) of *C. viatorum* were identified in Edwardʼs long-tailed rats in Hainan Province, China. However, these subtypes of *C. viatorum* were not found in the present investigation. The difference may be related to the geographical origin [22], the health status of the animals at the time of sampling, and the overall sample sizes.

A previous study [15] and the present study provide strong evidence that *C. viatorum* infection is prevalent in wild rats in China, suggesting that wild rats infected with *C. viatorum* may pose a threat to human health. Importantly, the *C. viatorum* XVa subtype family identified in the present study has significant public health implications, suggesting that these animals represent a potential zoonotic risk for the transfer of the pathogen in China. More in-depth studies are needed to understand the transmission of this *C. viatorum* XVa subtype family in rats. In addition, we believe that an increase in the number of wild rats from broader geographical locations and diversity of rat species surveyed for *C. viatorum* may help gain a much-improved understanding of the role of wild rats in the zoonotic transmission of *C. viatorum.*

## Conclusions

The present results indicate an occurrence of *C. viatorum* XVa subtype family in rats which are genetically identical to those found in humans. Our findings suggest that *L. edwardsi* and *B. bowersi* may play a potential role in the transmission of *C. viatorum* to other animals and humans. Future studies investigating the molecular epidemiology of *C. viatorum* in wild rats are needed to better clarify the risks and modes of transmission.

## Data Availability

The small subunit ribosomal RNA gene and gp60 gene sequences of *Cryptosporidium viatorum* from the wild rats have been deposited in the GenBank database under the accession numbers MK522269–MK522270 and MK796003–MK796005.

## Abbreviations

AIDS: Acquired Immune Deficiency Syndrome
BS: bootstrap support
BLAST: Basic Local Alignment Search Tool
CI: confidence interval
*cox*1: cytochrome *c* oxidase subunit 1
gp60: 60-kilodalton glycoproteingene
HIV: Human Immunodeficiency Virus
NJ: neighbor-joining
OR: odds ratio
PCR: polymerase chain reaction
*SSU* rRNA: small subunit ribosomal RNA
